# Deepening Our Understanding of COVID-19 Vaccine Decision-Making amongst Healthcare Workers in Southwest Virginia, USA Using Exploratory and Confirmatory Factor Analysis

**DOI:** 10.3390/vaccines11030556

**Published:** 2023-02-27

**Authors:** Jesse Bendetson, Mandy C. Swann, Alicia Lozano, Jennifer West, Alexandra L. Hanlon, Ian Crandell, Maimuna Jatta, Charles J. Schleupner, Anthony Baffoe-Bonnie

**Affiliations:** 1 Virginia Tech Carilion School of Medicine, Roanoke, VA 24016, USA; 2 Infection Prevention and Control Section, Carilion Clinic, Roanoke, VA 24014, USA; 3 Virginia Tech Center for Biostatistics and Health Data Science, Roanoke, VA 24016, USA; 4 Section of Infectious Diseases, Carilion Clinic, Roanoke, VA 24014, USA; 5 Department of Medicine, Carilion Clinic, Roanoke, VA 24014, USA

**Keywords:** vaccine, hesitancy, healthcare workers, COVID-19, psychometric constructs, factor analysis

## Abstract

Vaccine hesitancy amongst healthcare workers (HCWs) has been a major challenge throughout the COVID-19 pandemic. While many studies have identified HCW characteristics and specific attitudes associated with COVID-19 vaccine hesitancy, researchers are still working towards developing a holistic understanding of the psychological constructs that influence COVID-19 vaccine decision-making in this population. Between 15 March and 29 March 2021, we distributed an online survey assessing individual characteristics and vaccine-related perceptions to employees of a not-for-profit healthcare system in Southwest Virginia (N = 2459). We then performed exploratory factor analysis (EFA) and confirmatory factor analysis (CFA) to describe patterns of vaccine-related thought amongst HCWs and identify latent psychometric constructs involved in vaccine decision-making. The goodness of model fit was assessed using the Tucker–Lewis Index (TLI), the Comparative Fit Index (CFI), and the Root Mean Square Error of Approximation (RMSEA). Internal consistency and reliability of each factor were assessed using Cronbach’s alpha. EFA identified four latent psychometric constructs: Lack of trust in the COVID-19 vaccine; Anti-science sentiment; Adverse side-effects; and Situational risk assessment. The goodness of EFA model fit was adequate (TLI > 0.90, RMSEA ≤ 0.08) with acceptable internal consistency and reliability for three of four factors (Cronbach’s alpha > 0.70). The CFA model also had adequate goodness of fit (CFI > 0.90, RMSEA ≤ 0.08). We believe the psychometric constructs identified in this study can provide a useful framework for interventions to improve vaccine uptake amongst this critical population.

## 1. Introduction

As of 21 January 2023, SARS-CoV-2 has infected over 101.8 million individuals in the United States, resulting in just under 1.1 million deaths [1]. Four vaccines (Pfizer-BioNTech, Moderna, Johnson & Johnson, and Novavax) have been authorized for use in the US. Despite the widespread availability of these vaccines across the country and their demonstrated safety and efficacy [2,3,4,5], only 69.1% of Americans have completed the primary vaccine series, and just 15.3% have received their bivalent booster [1].

Anti-vaccine sentiment and vaccine hesitancy have been considerable public health challenges since long before the advent of COVID-19. The World Health Organization (WHO) declared vaccine hesitancy to be one of the top ten biggest threats to global health in 2017 [6], and it remains a major international concern that has hampered efforts to control the spread of COVID-19 [7]. Achieving widespread COVID-19 vaccination has become even more complicated by the politically charged nature of the issue, contributing to higher rates of hesitancy for some [8]. In addition, the novelty of mRNA vaccine technology and the speed with which these vaccines were developed may have further intensified hesitancy [9]. This matter is particularly problematic within the United States; a global survey of 17 countries from early in the COVID-19 vaccine rollout found that the US had the second highest proportion of respondents who said they would be unlikely to be vaccinated [10]. Common reasons for COVID-19 vaccine hesitancy included concerns regarding safety, efficacy, and both long- and short-term side-effects. Additionally, both female sex and lower educational attainment have been found to be predictive of hesitancy [11,12].

One group amongst whom COVID-19 vaccine hesitancy has been especially problematic over the course of the pandemic is healthcare workers (HCWs). Studies of HCWs in various contexts found degrees of hesitancy that ranged between 15% and 30% [13,14,15,16,17,18,19]. Demographic factors associated with COVID-19 vaccine hesitancy amongst HCWs included younger age [13,15,16,20,21,22,23], female sex [13,16,17,18,19,21,24,25,26,27], African American race [15,16,17,26,27], and lower educational attainment [17,23,27]. Role in healthcare has also been found to be predictive of vaccine hesitancy, with nurses consistently displaying higher levels of hesitancy compared to physicians [13,15,16,17,18,21,23,24,25,26,27,28]. Common reported reasons for COVID-19 vaccine hesitancy included concerns regarding safety [11,14,19,27,29], efficacy [11,16,17,27,29], fertility [13,15,17], and side-effects [11,13,14,15,16,26], as well as a desire to wait for more data [17,18,19,22,27]. Additionally, data show that more than 25% of HCWs may be hesitant to receive a booster dose [30,31].

While many studies have identified HCW characteristics and specific attitudes that are associated with COVID-19 vaccine hesitancy, to our knowledge, no research performed to present has measured the underlying psychological constructs associated with COVID-19 vaccine decision-making in this population. To fill this gap in the literature, we conducted exploratory and confirmatory factor analyses (EFA/CFA) on data from an internet-based survey of HCWs to characterize these latent constructs.

Factor analysis encompasses a set of analytical techniques that allow researchers to identify latent variables, or “factors,” that lie hidden within a dataset. By examining patterns of interrelationships between a dataset’s observed variables, investigators can characterize factors that cannot be measured directly but which may have broader implications than any individual measured variable has on its own [32].

EFA and CFA have been used to investigate the psychology underlying a wide variety of health behaviors. In their research on oral health practices, Xiang et al. identified six relevant psychometric factors based on the Health Belief Model: perceived susceptibility, perceived benefits, perceived barriers, cues to action, perceived severity, and self-efficacy [33]. Wang et al. determined that benefits, barriers, peer support, and self-efficacy are key factors that contribute to the psychology of medication non-adherence [34]. EFA and CFA have also been employed to deepen researchers’ understanding of food literacy behaviors in university students [35], pre-exposure prophylaxis adherence amongst HIV patients [36], and picky eating in children [37].

This article builds upon previously published research that identified demographic factors and individual beliefs associated with COVID-19 vaccine hesitancy amongst HCWs [16]. The present study employs EFA and CFA to identify and describe latent psychometric constructs that were involved in vaccine decision-making early in the vaccine roll-out. While vaccine mandates have required many HCWs to receive their initial vaccinations, remaining up to date with boosters will continue to be important to prevent COVID-19 infections in this population. Additionally, new, Omicron-adapted bivalent mRNA vaccines were authorized in August 2022 and are now recommended as booster doses [38]. As COVID-19 vaccinations and recommendations continue to evolve, and as we transition into an endemic phase of the pandemic, we believe the constructs characterized herein can provide a framework and efficient targets for interventions designed to improve vaccine and booster uptake amongst this critical population.

## 2. Materials and Methods

### 2.1. Sample and Study Design

Detailed study methods, including survey design and participant recruitment, have been described elsewhere [16]. Between 15 March and 29 March 2021, all 13,690 adult employees of a not-for-profit healthcare system in Southwest Virginia received an email with a link to our online questionnaire. Other distribution channels included a posting on the healthcare system intranet and print advertisements located in various clinical sites, which included QR codes for easy access.

Our survey was built in REDCap (Research Electronic Data Capture), a HIPAA-compliant web application and back-end database model developed at Vanderbilt University [39]. Participants could self-administer the questionnaire online using any device of their choosing. The survey instrument was written in English and was composed of 30 items addressing COVID-19 vaccination status, vaccine intentions, personal experiences with COVID-19, thoughts and beliefs regarding COVID-19 vaccines, and sociodemographic variables. The survey contained 15 five-point Likert-type items assessing level of agreement with various vaccine-related perceptions. The responses to four Likert-type items were reverse coded to scale all questions in the same direction, such that all higher scores indicated anti-vaccine sentiment and all lower scores indicated pro-vaccine sentiment. Respondents who had already received either one or two vaccine doses when they completed the questionnaire were categorized as vaccine acceptant. Because the COVID-19 vaccine had been available and accessible to respondents for over three months when we launched our survey, HCWs who had not received at least one vaccine dose at the time of survey completion were considered vaccine hesitant. The complete survey instrument is available in Supplement S1.

We received 2720 total survey responses, out of which 261 (9.6%) had missing information for at least one survey item. Differences between participants with and without missing information were deemed negligible due to small effect sizes (Cramer’s V < 0.40). We thus conducted a complete case analysis and analyzed information only from respondents with complete data on all survey items (N = 2459). The study sample was randomly divided into two groups; Group 1 (n = 1229) was used for exploratory factor analysis (EFA) and Group 2 (n = 1230) was used for confirmatory factor analysis (CFA).

### 2.2. Statistical Methods

Sociodemographic and work-related characteristics, as well as survey items pertaining to vaccine-related perceptions, were examined using descriptive statistics. The extraction of latent psychometric constructs that influenced COVID-19 vaccine decision-making was performed in Group 1 using EFA, where an oblique rotation (Oblimin) was used to facilitate the interpretation of the factors. Various sources of information were used to determine the number of factors to extract, including scree plots, factor diagrams, and subject matter expertise. The adequacy of the EFA model was assessed using the Tucker–Lewis Index (TLI), the Comparative Fit Index (CFI), and the Root Mean Square Error of Approximation (RMSEA). A CFI or TLI score ≥ 0.90 and a RMSEA < 0.08 indicated good fit [40]. Validation of the factor structure was then examined using CFA in Group 2, where adequate model fit was indicated by correlation residuals < 0.30. Finally, internal consistency and reliability of the factors were evaluated based on Cronbach’s alpha, where values of 0.70 or higher were considered acceptable [41]. All analyses were performed in R using the ‘lavaan’ package [42,43].

## 3. Results

### 3.1. Descriptive Statistics

Demographic and other HCW characteristics are presented in Table 1. Respondents were predominantly female (82%), white or Caucasian (89%), and aged 25 years or older (95%). The most represented healthcare role was nursing staff (34%), and the least represented was healthcare providers (8%). The distribution of responses to individual survey items is presented in Table 2.

### 3.2. Exploratory Factor Analysis (EFA)

**Figure A1:**
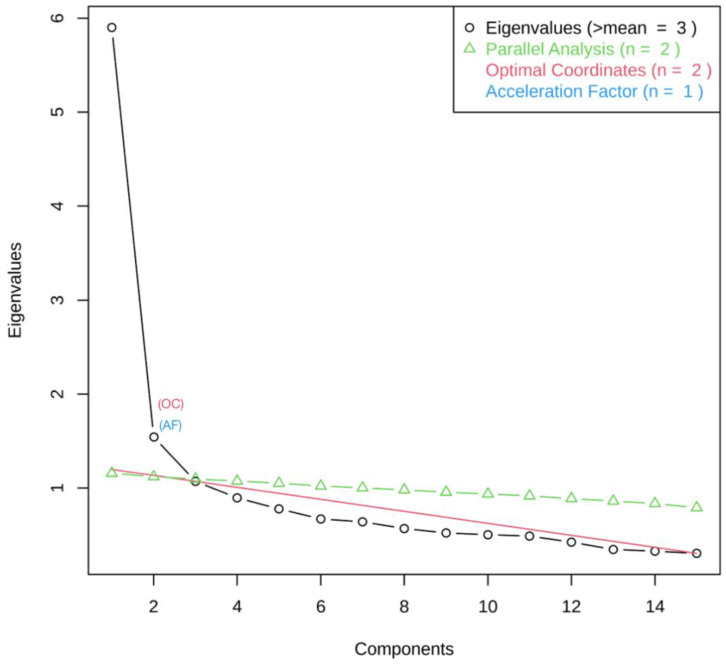
EFA Scree Plot.

Combined with scree plots (see Figure A1), factor diagrams, and subject matter expertise, EFA revealed four distinct factors influencing COVID-19 vaccine decision-making amongst HCWs:

- Factor 1: Lack of trust in the COVID-19 vaccine
- Factor 2: Anti-science sentiment
- Factor 3: Adverse side-effects
- Factor 4: Situational risk assessment

**Figure A2:**
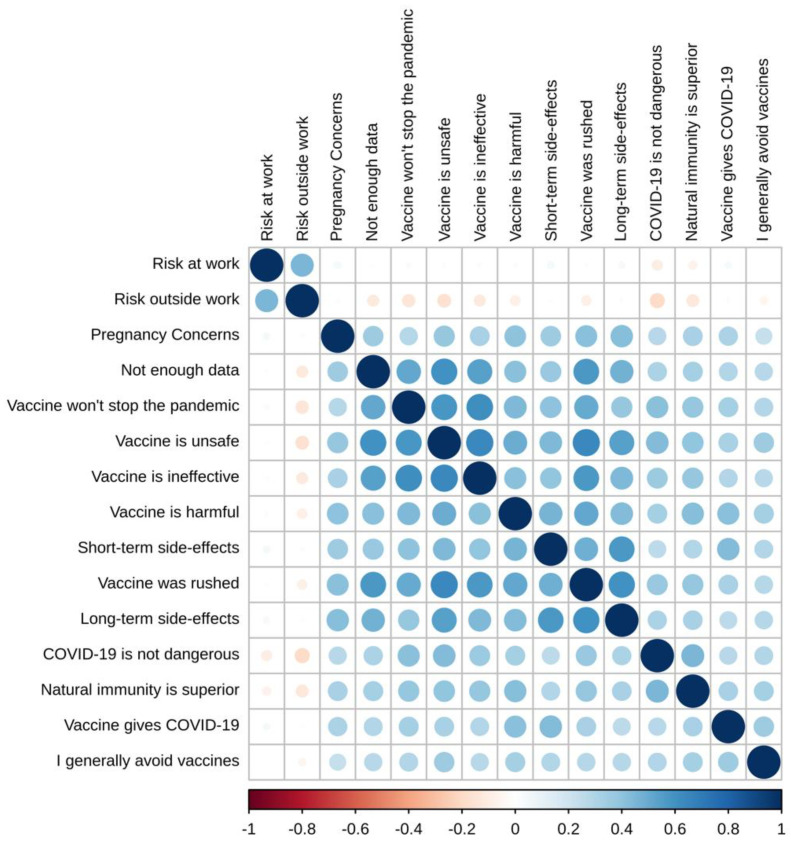
Graphical Presentation of Correlations Between EFA Factors.

Correlations between factors are presented in Table A1 (see Exploratory Factor Analysis) and Figure A2 in Appendix A. Although Factor 4 was weakly correlated with the other three factors, it was retained in our model because excluding the items that loaded onto this factor (Work Risk and Outside Risk) produced a model that did not yield meaningful psychometric constructs. Table 3 contains the survey items that comprised each factor, the spread of the data that was accounted for by each factor (i.e., the proportion of variance explained), and a measure of each factor’s internal consistency and reliability (Cronbach’s alpha). All survey items had strong or moderate correlations with their respective factors. The largest proportion of the variance (40.7%) was accounted for by Factor 1 (Lack of trust in the COVID-19 vaccine). The EFA model demonstrated a good fit to the data distribution (CFI = 0.932; RMSEA = 0.07).

### 3.3. Confirmatory Factor Analysis (CFA)

**Figure 1:**
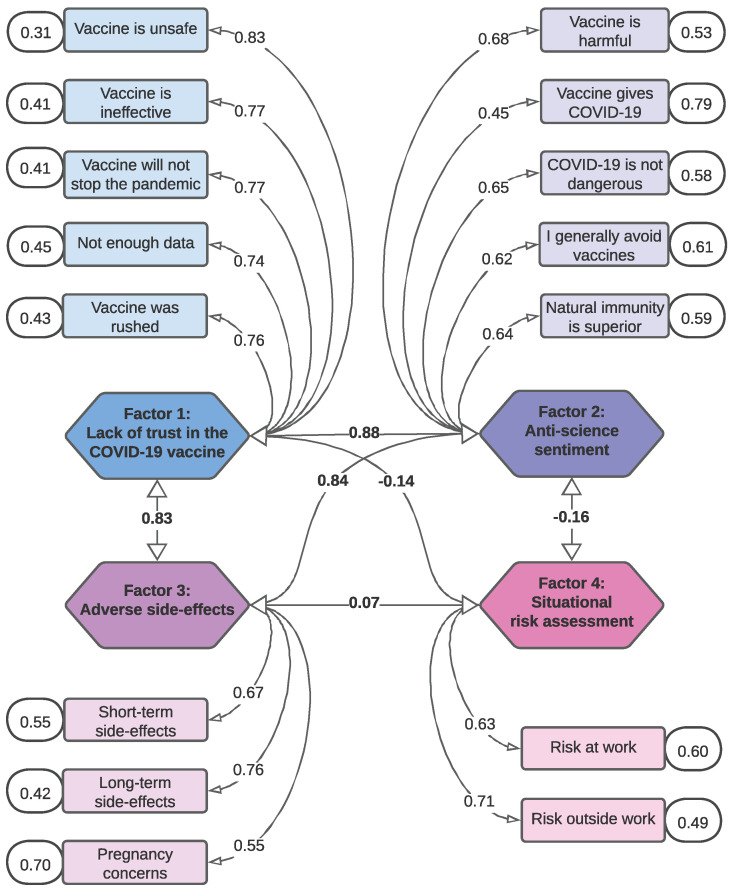
CFA Factor Diagram. Survey items mapped to the factors onto which they loaded most strongly in CFA. The hexagons in the center represent factors, with each arrow from factor to factor providing the standardized covariance (Pearson’s correlation, r) between those two factors. The rectangles represent survey items, with each arrow from factor to survey item providing the factor loading (correlation between factor and survey item) for the factor onto which that survey item loaded most strongly. The ovals in the outer margins next to the survey items provide the variance of each individual survey item.

CFA on the four-factor model showed a good fit to the data as evidenced by weak residual correlations (<0.30). The CFA model is visualized in Figure 1, where the survey items and the extracted factors onto which they loaded most strongly are presented along with the factor loadings (i.e., the correlation of each survey item with its associated factor) and correlations between factors (see Table A1, Confirmatory Factor Analysis). All survey items had strong correlations with their respective factors. Except Factor 4 (Situational risk assessment), which consisted of only two survey items (Cronbach’s alpha = 0.637), all factors demonstrated good internal consistency and reliability with a Cronbach’s alpha > 0.70 (Table 3).

## 4. Discussion

By assessing a broad range of individual beliefs and perceptions, we identified four underlying psychological constructs associated with COVID-19 vaccine decision-making amongst HCWs: Lack of trust in the COVID-19 vaccine; Anti-science sentiment; Adverse side-effects; and Situational risk assessment. Much of the prior research on predictors of COVID-19 vaccine uptake amongst HCWs focused on specific characteristics, attitudes, and opinions associated with vaccine hesitancy. By employing EFA and CFA techniques to extract and validate latent psychometric constructs, we have described broader patterns of thought related to HCW COVID-19 vaccine decision-making.

The psychometric factors we identified share considerable overlap with the 5C psychological antecedents of vaccination, a model that identifies Confidence, Constraints, Complacency, Calculation, and Collective responsibility as contributors to vaccine hesitancy [44]. Elements of the 5C model have been explored as predictors of COVID-19 vaccine hesitancy amongst HCWs in Hong Kong [20], Singapore [30], and Kuwait [45]. The 5C model has also more recently been expanded to a 7C model with the additions of Conspiracy and Compliance [46]. We feel that this manuscript builds on such research by analyzing data from a survey that was expressly developed with COVID-19 vaccines in mind and which may thus hone in more closely on the specific concerns that are relevant to vaccination in the context of a rapidly evolving pandemic. Additionally, both the 5C and 7C models were initially tested on general population convenience samples [44,46], while the present analysis was performed on a convenience sample comprised entirely of HCWs; the factor structure identified herein may thus more closely reflect the psychometric constructs that influence vaccine decision-making in this specific population.

While vaccine mandates have required many healthcare workers to receive their initial COVID-19 vaccines irrespective of individual hesitancy, epidemiologic data demonstrate that staying up to date on booster doses provides the most effective protection over time [47]. Despite such evidence, a survey from 2022 found that 87.3% of HCWs reported finishing their primary vaccine series, but just 67.3% reported receiving a booster dose [48]. More recent data from the CDC show that although 86.4% of nursing home staff in the United States completed their primary vaccination series, only 22.6% remain up to date on their COVID-19 vaccines [49]. Strategies that combat booster hesitancy will thus be essential to protecting the healthcare workforce and supporting robust healthcare systems in the future. Because our data were collected when COVID-19 vaccines were very new, we believe our findings will remain relevant moving forward as HCWs periodically decide whether they will get the latest iteration in an evolving series of new vaccines and booster doses. The psychometric factors identified in our study can provide a theoretical framework and discrete areas of emphasis for nuanced messaging and effective interventions to support vaccine coverage in this high-priority population. Specifically, our results suggest that strategies that build trust in these newer vaccines (e.g., by focusing on safety and efficacy concerns) will address underlying hesitancy and may be the most successful at improving uptake among HCWs. Similarly, interventions that alleviate fears about adverse vaccine-related outcomes (e.g., pregnancy complications, and long-term side-effects) may influence decision-making and reduce hesitancy to adopt new, bivalent mRNA vaccines and boosters.

Prior research has shown the need for nuanced strategies to address the multifactorial causes of vaccine hesitancy amongst a diverse healthcare workforce [50]. Numerous studies have investigated interventional strategies to improve COVID-19 vaccine uptake in the early days of vaccine availability. A brief educational video focused on vaccine characteristics, development, side-effects, conspiracy theories, and public health guidelines enhanced COVID-19 vaccine-related thoughts and perceptions amongst a representative sample of Israeli adults [51]. A physician-led, in-person informational intervention centered around the impact of COVID-19, mRNA vaccines (mechanism of action, clinical trial data, adverse events), and common vaccine myths and concerns decreased hesitancy amongst a sample of US active-duty military members [52]. A multifaceted educational program delivered to HCWs at a large tertiary-care center in Japan that included information pamphlets, hospital-wide announcements encouraging COVID-19 vaccination, and mandatory information sessions was associated with increased vaccine uptake [53]. Our research provides a theoretical basis for the successful results observed in these studies, as each of these interventions focused primarily on the science surrounding the safety, efficacy, and side-effects of the newly available COVID-19 vaccines. While our findings also indicate that general anti-science sentiment plays a role in COVID-19 vaccine decision-making in this context, research suggests that this construct may require more complex persuasive strategies to overcome [54].

This study has several limitations. First, we used a convenience sample of survey respondents, and our results may, therefore, not generalize to other HCWs in this health system; additionally, our narrow geographic focus may limit generalizability to HCWs in other settings. Second, although our survey was self-administered and respondents were frequently reminded that their information would be deidentified throughout the questionnaire, the survey was distributed through work channels, and as a result, our data may include some degree of social desirability bias. Third, all responses were received during a two-week period in March 2021. While our results reflect factors associated with decision-making regarding COVID-19 vaccines when they first became available, our data may not directly mirror the thoughts and perceptions that will emerge related to the novelty of adapted vaccines. However, because the psychometric factors identified herein reflect responses to the initial COVID-19 vaccines when they were still very new, we feel our results will remain relevant as novel vaccines and boosters are released and as vaccine technology continues to develop going forward.

Future plans for these data include logistic regression analyses and multidimensional modeling to determine whether the latent constructs we identified are directly predictive of vaccine hesitancy.

With more than 25% of HCWs hesitant to receive a booster dose and with booster uptake flagging in the United States [1], there is a dire need to reduce hesitancy amongst HCWs with unavoidable occupational exposure risk [30,31]. We believe our findings provide a roadmap for developing interventions that can combat uncertainties around COVID-19 vaccines and booster doses—including new bivalent mRNA vaccines – amongst this critical population.

## Figures and Tables

**Table 1:** Demographic and Other Healthcare Worker Characteristics (N = 2459).

**Characteristic**	**n (%)**
Sex	
Female	2019 (82.1%)
Male	423 (17.2%)
Non-Binary	5 (0.2%)
Prefer Not to Say	12 (0.5%)
Age Group	
18–24	114 (4.6%)
25–34	560 (22.8%)
35–44	545 (22.2%)
45–54	613 (24.9%)
55–64	545 (22.2%)
65+	82 (3.3%)
Race/Ethnicity	
Asian	41 (1.7%)
Black/ African American	127 (5.2%)
Hispanic/Latino(a)	39 (1.6%)
Native American or Alaska Native	16 (0.7%)
Native Hawaiian or Other Pacific Islander	1 (0.04%)
White/Caucasian	2181 (88.7%)
Two or More Races	32 (1.3%)
Unknown	22 (0.9%)
Role at Carilion *	
Nursing	827 (33.6%)
Provider	194 (7.9%)
Management	280 (11.4%)
Other Responsibilities (Patient Care)	605 (24.6%)
Other Responsibilities (Non-Patient Care)	553 (22.5%)
Work Setting	
Outpatient	767 (31.2%)
Inpatient	696 (28.3%)
Both	464 (18.9%)
Other	490 (19.9%)
Unsure	42 (1.7%)
Hesitancy Status	
Vaccine Hesitant	446 (18.1%)
Vaccine Acceptant	2013 (81.9%)

∗ “Nursing” includes nurses, nursing assistants, clinical associates, and nursing management. "Provider" includes physicians, physician assistants, and nurse practitioners. “Management” includes managers without patient care responsibilities. “Other Responsibilities (Patient Care)” includes staff that have responsibilities in patient care areas. “Other Responsibilities (Non-Patient Care)” includes staff whose work is conducted outside of patient care areas.

**Table 2:** Survey Item Responses (N = 2459).

**Survey Item**	
Vaccine is ineffective	
Mean (SD)	2.20 (±1.02)
Median [Min, Max]	2 [1, 5]
Vaccine is unsafe	
Mean (SD)	2.19 (±1.15)
Median [Min, Max]	2 [1, 5]
Vaccine won’t stop the pandemic	
Mean (SD)	2.07 (±1.07)
Median [Min, Max]	2 [1, 5]
Vaccine was rushed	
Mean (SD)	2.48 (±1.25)
Median [Min, Max]	2 [1, 5]
Vaccine gives COVID-19	
Mean (SD)	1.32 (±0.74)
Median [Min, Max]	1 [1, 5]
Natural immunity is superior	
Mean (SD)	2.30 (±1.11)
Median [Min, Max]	2 [1, 5]
I generally avoid vaccines	
Mean (SD)	1.62 (±1.06)
Median [Min, Max]	1 [1, 5]
Vaccine is harmful	
Mean (SD)	2.00 (±1.10)
Median [Min, Max]	2 [1, 5]
COVID-19 is not dangerous	
Mean (SD)	1.45 (±0.86)
Median [Min, Max]	1 [1, 5]
Long-term side-effects	
Mean (SD)	3.09 (±1.31)
Median [Min, Max]	3 [1, 5]
Short-term side-effects	
Mean (SD)	2.23 (±1.21)
Median [Min, Max]	2 [1, 5]
Pregnancy concerns	
Mean (SD)	1.98 (±1.18)
Median [Min, Max]	1 [1, 5]
Risk at work	
Mean (SD)	3.00 (±1.29)
Median [Min, Max]	3 [1, 5]
Risk outside work	
Mean (SD)	2.96 (±1.13)
Median [Min, Max]	3 [1, 5]

1 = Strongly Disagree, 2 = Somewhat Disagree, 3 = Neither Agree Nor Disagree, 4 = Somewhat Agree, 5 = Strongly Agree.

**Table 3:** Factor Loadings and Internal Reliability.

Survey Item	Factor 1	Factor 2	Factor 3	Factor 4
Vaccine is ineffective	0.827	–	–	–
Vaccine is unsafe	0.717	–	–	–
Vaccine won’t stop the pandemic	0.709	–	–	–
Not enough data	0.615	–	–	–
Vaccine was rushed	0.492	–	0.387	–
Vaccine gives COVID-19	–	0.633	–	–
Natural immunity is superior	–	0.503	–	–
I generally avoid vaccines	–	0.499	–	–
Vaccine is harmful	–	0.457	–	–
COVID-19 is not dangerous	–	0.373	–	–
Long-term side-effects	–	–	0.694	–
Short-term side-effects	–	0.339	0.491	–
Pregnancy concerns	–	–	–	–
Risk at work	–	–	–	0.686
Risk outside work	–	–	–	0.677
Proportion of Variance Explained	0.407	0.259	0.202	0.133
Number of Survey Items	5	5	3	2
Cronbach’s Alpha	0.880	0.733	0.701	0.637

Factor 1: Lack of trust in the COVID-19 vaccine; Factor 2: Anti-science sentiment; Factor 3: Adverse side-effects; Factor 4: Situational risk assessment.

## Data Availability

The data from this study may be obtained upon email request to the senior author, Anthony Baffoe-Bonnie (awbaffoebonnie@carilionclinic.org).

## Supplementary Materials

The following supporting information can be downloaded at: https://www.mdpi.com/article/10.3390/vaccines11030556/s1, Supplement S1: Survey Instrument.

## Abbreviations

The following abbreviations are used in this manuscript:

HCW	Healthcare Worker
EFA	Exploratory Factor Analysis
CFA	Confirmatory Factor Analysis
TLI	Tucker Lewis Index
CFI	Comparative Fit Index
RMSEA	Root Mean Square Error of Approximation

## Appendix A

**Table A1:** Correlation Matrix of Factors.

	Exploratory Factor Analysis				Confirmatory Factor Analysis			
	**Factor 1**	**Factor 2**	**Factor 3**	**Factor 4**	**Factor 1**	**Factor 2**	**Factor 3**	**Factor 4**
Factor 1: Lack of trust in the COVID-19 vaccine	1.000	0.767 ***	0.724 ***	−0.211 ***	1.000	0.882 ***	0.835 ***	−0.142 **
Factor 2: Anti-science sentiment	0.767 ***	1.000	0.660 ***	−0.143 ***	0.882 ***	1.000	0.843 ***	−0.163 **
Factor 3: Adverse side-effects	0.724 ***	0.660 ***	1.000	0.041	0.835 ***	0.843 ***	1.000	0.066
Factor 4: Situational risk assessment	−0.211 ***	−0.143 ***	0.041	1.000	−0.142 **	−0.163 **	0.066	1.000

Note: Pearson’s correlation coefficients (r) are interpreted as small (0.10–0.29), moderate (0.30–0.49), and large (0.50–1.0). * *p* < 0.05; ** *p* < 0.01; *** *p* < 0.001.
