# Supplementary material for: Deepening Our Understanding of COVID-19 Vaccine Decision-Making amongst Healthcare Workers in Southwest Virginia, USA Using Exploratory and Confirmatory Factor Analysis
Source: Vaccines (Basel). 2023 Feb 27;11(3):556. doi: 10.3390/vaccines11030556 (PMC10058545; doi:10.3390/vaccines11030556)
Supplement: Supplementary file 1 [file vaccines-11-00556-s001.zip › vaccines-2123658-supplementary.pdf]

Supplement S1 – Survey Instrument

We are conducting a brief survey of all Carilion Clinic employees to understand their feelings about the COVID-19 vaccine. There are currently three COVID-19 vaccines approved by the FDA, developed by Pfizer, Moderna, and Johnson & Johnson. All Carilion Clinic employees have been offered a COVID-19 vaccine.

This brief survey will take about 5 minutes to complete. Please answer all questions and provide your open and honest input.

Your answers are confidential and will not be linked to your name or employee record in any way.

	Yes	No	Unsure
**Have you had COVID-19 before?**	◯	◯	◯
**Have you had a family member or loved one become ill due to COVID-19?**	◯	◯	◯
**Have you had a family member or loved one die as a result of COVID-19?**	◯	◯	◯
**Is there anyone living in your household who might be considered high-risk for a bad outcome?**	◯	◯	◯

Have you already received a vaccine for COVID-19?

◯ Yes, one dose
◯ Yes, both doses
◯ Yes, I got a single-dose vaccine
◯ No

If VACCINATED = 'Yes, one dose'
Do you plan to get your second dose?

◯ Yes
◯ No
◯ Unsure

If VACCINATED = 'No'
Do you intend to get the COVID-19 vaccine in the near future?

◯ Yes
◯ No
◯ Unsure

Below are several statements that reflect views about the COVID-19 vaccine. Please select your level of agreement with each statement from Strongly Disagree (1) to Strongly Agree (5).

(1) = Strongly Disagree
(2) = Somewhat Disagree
(3) = Neither Agree Nor Disagree
(4) = Somewhat Agree
(5) = Strongly Agree

	(1)	(2)	(3)	(4)	(5)
**I believe I am at risk of getting COVID-19 while at work**	○	○	○	○	○
**I believe I am at risk of getting COVID-19 outside of work**	○	○	○	○	○
**COVID-19 is not that dangerous so I do not need a vaccine**	○	○	○	○	○
**I believe the COVID-19 vaccine is safe**	○	○	○	○	○
**I feel that COVID-19 vaccine development was rushed without sufficient quality control**	○	○	○	○	○
**I believe the COVID-19 vaccine is effective in preventing COVID-19**	○	○	○	○	○
**I am worried about the known/reported short-term side-effects from the COVID-19 vaccine (e.g., fever, fatigue, headache)**	○	○	○	○	○
**I am worried about the potential (unknown) long-term side-effects of the COVID-19 vaccine**	○	○	○	○	○
**I am worried that the vaccine will give me COVID-19**	○	○	○	○	○
**I feel there is enough information/data about the COVID-19 vaccine to make an informed decision**	○	○	○	○	○
**I believe natural immunity from getting COVID-19 is more protective than a vaccine**	○	○	○	○	○

**I am concerned about information I have seen that the vaccine is very harmful**	◯	◯	◯	◯	◯
**I believe the COVID-19 vaccine will help stop the pandemic**	◯	◯	◯	◯	◯
**In general, I try to avoid vaccinations**	◯	◯	◯	◯	◯
**I am worried the vaccine will negatively affect a current pregnancy or my future ability to have children**	◯	◯	◯	◯	◯

If VACCINATED = 'No' and INTEND TO VACCINATE = 'No' or 'Unsure'
What is the MOST IMPORTANT reason you have not gotten the COVID-19 vaccine? (Choose the best answer.)

◯ I am not at risk for COVID-19
◯ I have already had COVID-19 so I do not need it
◯ I have a medical condition that prevents me from getting it until there is more data
◯ I have not had time
◯ I have concerns about vaccine safety
◯ I have seen information that the vaccine is harmful
◯ I am worried about vaccine side-effects (fever, fatigue, headache, etc.)
◯ I do not think the vaccine is effective
◯ I worry the vaccine will give me COVID-19
◯ I try to avoid all vaccines
◯ Other – Specify

If VACCINATED = 'No' and INTEND TO VACCINATE = 'Yes'
What is the MOST IMPORTANT reason you have not yet gotten the COVID-19 vaccine? (Choose the best answer.)

◯ I am not at risk for COVID-19
◯ I have already had COVID-19 so I do not need it
◯ I have a medical condition that prevents me from getting it until there is more data
◯ I have not had time
◯ I have concerns about vaccine safety
◯ I have seen information that the vaccine is harmful
◯ I am worried about vaccine side-effects (fever, fatigue, headache, etc.)
◯ I do not think the vaccine is effective
◯ I worry the vaccine will give me COVID-19
◯ I try to avoid all vaccines

◯ Other – Specify

If VACCINATED = 'Yes, one dose,' 'Yes, two doses,' or 'Yes, I got a single-dose vaccine'
If you hesitated at all before receiving the COVID-19 vaccine, what was your MAIN reservation? (Choose the best answer.)

◯ I am not at risk for COVID-19
◯ I have already had COVID-19 so I do not need it
◯ I have a medical condition that prevents me from getting it until there is more data
◯ I have not had time
◯ I have concerns about vaccine safety
◯ I have seen information that the vaccine is harmful
◯ I am worried about vaccine side-effects (fever, fatigue, headache, etc.)
◯ I do not think the vaccine is effective
◯ I worry the vaccine will give me COVID-19
◯ I try to avoid all vaccines
◯ Other – Specify

If VACCINATED = 'Yes, one dose,' 'Yes, two doses,' or 'Yes, I got a single-dose vaccine'
What was the MOST IMPORTANT reason you decided to get the COVID-19 vaccine? (Choose the best answer.)

◯ I am at risk of getting COVID-19 at work
◯ I am at risk of getting COVID-19 outside of work
◯ It will allow me to go back to doing the things I used to without worry
◯ I have a medical condition that puts me at risk for severe disease
◯ I have a family member that I want to protect
◯ I want to do my part to help end the pandemic
◯ I have seen information that the vaccine is safe
◯ I believe the risk of side-effects is outweighed by the benefits of the vaccine
◯ I typically always take vaccines
◯ Other – Specify

Where do you get trustworthy information about COVID-19? (Select all that apply.)

☐ Personal Physician
☐ Family and friends
☐ Coworkers
☐ Supervisor/Unit Director
☐ Carilion Management/Inside Carilion

☐ News Media (Newspapers, Online News Sites, TV, Radio)
☐ Social Media (Facebook, Twitter, Instagram, TikTok, YouTube, etc.)
☐ Other – Specify

Which of the following would you use to describe yourself?

◯ Female
◯ Male
◯ Non-Binary
◯ Prefer to Self-Describe – Specify

Age

◯ <25
◯ 25-34
◯ 35-44
◯ 45-54
◯ 55-64
◯ 65+

How would you classify yourself in terms of race or ethnicity? (Select all that apply.)

☐ Asian
☐ Black or African American
☐ Native American or Alaska Native
☐ Native Hawaiian or Other Pacific Islander
☐ White or Caucasian
☐ Other – Specify

What is your role at Carilion Clinic?

◯ Provider (Physician, Physician Assistant, Nurse Practitioner)
◯ Nursing Staff (Nursing Management, Nurse, Nursing Assistant, Clinical Associate)
◯ Other Responsibilities in Patient Care Areas
◯ Other Responsibilities in Non-Patient Care Areas
◯ Management (No Clinical Care)

Do you work in an inpatient or outpatient setting?

◯ Inpatient
◯ Outpatient

◯ Both
◯ Unsure
◯ Other – Specify

In which county/city do you live?

◯ Alleghany County
◯ Augusta County
◯ Bath County
◯ Bedford County
◯ Bland County
◯ Botetourt County
◯ Craig County
◯ Floyd County
◯ Franklin County
◯ Giles County
◯ Montgomery County
◯ Nelson County
◯ Pulaski County
◯ Roanoke City
◯ Roanoke County
◯ Rockbridge County
◯ Salem
◯ Tazewell County
◯ Wythe County
◯ Unsure
◯ Other – Specify
